# Integrative analysis of cuproptosis in kidney ischemia-reperfusion injury: biomarker discovery and diagnostic model construction

**DOI:** 10.3389/fmolb.2026.1740289

**Published:** 2026-06-26

**Authors:** Chunyan Liang, Lixin Fu, Manling Xie, Changquan Zhang, Rong Ma, Chao Hu, Haibin Li, Ning Wen, Jiqiu Wen, Jianhui Dong, Xuyong Sun

**Affiliations:** 1 Institute of Transplant Medicine, The Second Affiliated Hospital of Guangxi Medical University, Nanning, China; 2 Guangxi Clinical Research Center for Organ Transplantation, Nanning, China; 3 Guangxi Key Laboratory of Organ Donation and Transplantation, Nanning, China

**Keywords:** bioinformatic analysis, cuproptosis, diagnostic models, immune cell infiltration, kidney ischemia-reperfusion injury

## Abstract

**Purpose:**

Acute kidney injury (AKI) is often associated with kidney ischemia-reperfusion injury (KIRI), which is a key driver of AKI progression. Although cuproptosis has been implicated in multiple pathological processes, its relevance to KIRI remains unclear. This study aimed to identify candidate cuproptosis-related genes associated with KIRI and characterize their potential diagnostic and biological relevance.

**Methods:**

Bioinformatic analyses of transcriptome datasets were performed to identify cuproptosis-related differentially expressed genes (CRDEGs) in KIRI. GSE43974 served as the discovery cohort, GSE126805 served as the external validation cohort, and GSE161201 was used for exploratory single-cell analysis. Focusing on these CRDEGs, we constructed a diagnostic model for KIRI using machine learning and validated it with a nomogram. Gene set enrichment analysis (GSEA), immune infiltration analysis, and correlation analyses were used to investigate signaling pathways and immune processes associated with CRDEGs. Unsupervised clustering was conducted to classify KIRI samples and characterize CRDEG-related molecular subtypes. CRDEG expression was further verified in a mouse KIRI model treated with cuproptosis modulators. Transcription factor–mRNA, miRNA–mRNA, and drug–gene interaction networks were constructed to explore potential regulatory and therapeutic associations.

**Results:**

Four CRDEGs (*PPP1R15A, LIPT1, SERPINE1*, and *HSPD1*) were identified. and associated with immune cell infiltration. Machine-learning analyses suggested the potential of these genes as candidate biomarkers for KIRI, and a nomogram based on these genes showed high diagnostic performance in the discovery cohort. These four CRDEGs showed higher expression in KIRI samples than in control samples. GSEA linked these genes to the immune response, oxidative stress, apoptosis-related signaling, and other injury-associated pathways. Cluster analysis revealed two KIRI subtypes (groups A and B) with differing molecular signatures and pathway activity, especially those related to apoptosis signaling, oxidative stress, and immune responses. Their potential diagnostic relevance was further assessed using external datasets and a KIRI animal model. In addition, 103 transcription factors, 152 microRNAs, and 36 drugs potentially interacting with these CRDEGs were predicted.

**Conclusion:**

*PPP1R15A, LIPT1, SERPINE1,* and *HSPD1* may serve as candidate indicators of the involvement of cuproptosis in KIRI, although further mechanistic validation is needed.

## 1 Introduction

Kidney ischemia‒reperfusion injury (KIRI) occurs when the restoration of disrupted renal blood flow paradoxically exacerbates renal dysfunction and tissue damage (Pefanis et al., 2019). In clinical settings, KIRI is a major contributor to acute kidney injury (AKI) and frequently occurs after renal transplantation. It substantially affects the prognosis of AKI, early functional recovery, and long-term graft survival after transplantation (Platt et al., 2022). KIRI is a complex disorder characterized by multiple molecular pathway alterations during pathogenesis (Vallés et al., 2014). Key mechanisms implicated in KIRI include free radical-induced injury, intracellular calcium overload, inflammatory responses, mitochondrial dysfunction, and apoptosis (Lv et al., 2024; Malek and Nematbakhsh, 2015). At present, effective clinical therapeutic approaches for KIRI remain limited, highlighting the need for further investigation into its molecular mechanisms and potential individualized therapeutic approaches.

Regulated cell death has emerged as an important mechanism and potential therapeutic target in KIRI (Liu and Liu, 2022). In 2022, cuproptosis was identified as a distinct form of copper-dependent programmed cell death (Tsvetkov et al., 2022). The occurrence of cuproptosis depends largely on mitochondrial metabolic activity, especially the operation of the TCA cycle. Under conditions of copper overload, copper ions bind to lipoylated mitochondrial enzymes involved in this cycle, which promotes abnormal protein aggregation and destabilizes iron–sulfur cluster proteins. This sequence of events eventually leads to proteotoxic stress and cell death (Tsvetkov et al., 2022). Copper is an essential micronutrient involved in mitochondrial energy production, biomolecule synthesis, and antioxidant defense (Yang et al., 2023; Garz et al., 2023). Abnormal copper accumulation or deficiency may disrupt cellular homeostasis and contribute to disease development. Disturbances in copper homeostasis frequently correlate with inherited conditions such as Wilson’s and Menkes syndromes, in addition to being associated with some malignancies and inflammatory disorders (Gao and Zhang, 2023; Lou et al., 2024).

Copper homeostasis is regulated mainly through intestinal absorption, hepatic storage and biliary excretion, and renal handling (Ge et al., 2022; Jiayi et al., 2024). The liver excretes excess copper ions into bile, and this copper is eventually eliminated from the body through feces (Shanbhag et al., 2021). When copper ion levels exceed the normal range, they can also be excreted through the kidneys into the urine. During copper deficiency, proximal tubule cells reabsorb copper from the filtrate. Copper ions can then be transported back into the circulation through copper-transporting ATPase. Recent studies have confirmed that early injury to renal tubular cells during KIRI can potentially trigger alterations in systemic copper homeostasis (Ye et al., 2021). Additionally, increased urinary copper concentrations might suggest a greater likelihood of AKI (Li et al., 2023). Emerging research further suggests possible associations between cuproptosis and additional kidney pathologies, including clear cell renal cell carcinoma and chronic kidney disease (Ji et al., 2022; Chen et al., 2024). Recent studies have begun to explore KIRI diagnostic models and relevant cuproptosis-related genes. Therefore, this study aimed to identify candidate cuproptosis-related genes associated with KIRI and evaluate their potential diagnostic and biological relevance through integrated transcriptomic analysis, experimental validation, and regulatory network prediction.

## 2 Materials and methods

### 2.1 Data acquisition and handling

Public transcriptomic datasets related to kidney transplantation (KTx) and KIRI were obtained from the GEO database (https://www.ncbi.nlm.nih.gov/geo/) (Barrett et al., 2012). Three datasets were included: GSE43974 was used as the discovery cohort, GSE126805 was used as the external validation cohort, and GSE161201 was used for exploratory single-cell analysis. (1) In the GSE43974 dataset: samples collected at the T1 stage (kidney biopsies obtained prior to organ retrieval from deceased donors), encompassing brain-dead (BD), donation after circulatory death (DCD), and living donor specimens, were designated as pre-ischemia controls. Conversely, samples procured at the T3 stage (60 min post-reperfusion), including those from BD, DCD, and living donors, were classified as post-reperfusion cases (KIRI group). In total, 188 samples were collected from the control group and 203 samples from the KIRI group (Damman et al., 2015). (2) GSE126805, including 42 pre-reperfusion and 41 post-reperfusion kidney biopsy samples, was used only for external validation (Cippà et al., 2018). (3) The GSE161201 dataset, derived from a mouse single-cell study, includes one control sample from a normal kidney and two samples collected at 6 h and 24 h post-reperfusion respectively, and was used for exploratory single-cell analysis (Ide et al., 2021). Collection of CRGs (Copper-Related Genes): By searching for “cuproptosis” on Gene Cards (https://www.genecards.org/) and NCBI Gene(https://www.ncbi.nlm.nih.gov/), and in conjunction with previously published literature, a total of 352 CRGs were collected (Xie et al., 2023; Tang et al., 2022).

### 2.2 Identification of DEGs associated with KIRI

Positive differentially expressed genes (DEGs) in the GSE43974 dataset were identified using the R package “limma” by applying the following thresholds: FDR < 0.05 and |log_2_FC| > 0.5 (Ritchie et al., 2015). Volcano plots and heatmaps of DEGs were created using the R packages “ggplot2” and “ComplexHeatmap” respectively (Meng et al., 2024). Subsequently, an intersection analysis of DEGs and CRGs was performed using the R package ‘Venn Diagram’, and these overlapping genes were defined as cuproptosis-related differentially expressed genes (CRDEGs). Furthermore, the R package ‘clusterProfiler’ was used to perform enrichment analysis, aiming to explore the biological functions and pathways linked to these genes. This analysis included Gene Ontology (GO) functional enrichment and Kyoto Encyclopedia of Genes and Genomes (KEGG) pathway analysis (Wu et al., 2021; Kanehisa et al., 2023).

### 2.3 Exploration of protein interactions of CRDEGs

A network of protein interactions was generated using the STRING platform (Szklarczyk et al., 2021), depicting connections between proteins produced by these four CRDEGs. The analysis employed the following parameters: human species designation, interaction score threshold of 0.400, and false discovery rate below 5%. The results of the analysis were visualized with Cytoscape software.

### 2.4 Construction and validation of a KIRI risk prediction model based on CRDEGs

Our study was based on an expression dataset containing 391 samples (203 in the IRI group and 188 in the non-IRI group), and systematic analyses were performed on four core genes (*PPP1R15A, LIPT1, SERPINE1,* and *HSPD1*). In this study, the IRI group represented post-reperfusion kidney biopsy samples, whereas the non-IRI group represented pre-ischemia samples. First, descriptive statistics and independent samples t tests were used to assess the expression levels and significance of differences in genes between groups. Subsequently, univariate and multivariate logistic regression analyses were performed separately. The pROC package (Robin et al., 2011) was used to plot the receiver operating characteristic (ROC) curves, and the predictive efficacy of the combination of single genes and multiple genes for IRI was assessed using the area under the curve (AUC). Furthermore, performance indexes such as model accuracy and sensitivity were calculated from the confusion matrix. In addition, the rms package was applied to construct nomogram column line graphs for quantitative prediction and visualization of the risk of IRI in individuals. Ultimately, the results were presented interactively via box-and-line plots, forest plots, and ROC curves. GSE126805 was used as an independent public GEO validation cohort and was not involved in feature selection, coefficient estimation, or model construction.

### 2.5 GSEA for CRDEGs

GSEA was performed using the R packages “limma” and ‘clusterProfiler’ for statistical computation (Yu et al., 2012). For each CRDEG, KIRI samples were divided into high- and low-expression subgroups based on the median gene expression level. Mean expression values were calculated for each subgroup and converted into log fold-change values. The average expression levels within each group were computed and then transformed into logFC values for further analysis. Gene set enrichment analysis was performed using the GSEA method, with a significance cutoff of p < 0.05. The most prominent ten pathways meeting this criterion were graphically represented through the gseaplot2 visualization tool.

### 2.6 Analysis of immune infiltration and inflammation-, apoptosis-, and proliferation-related factors

The CIBERSORT computational tool (https://cibersortx.stanford.edu/) was employed to examine immune cell composition in non-IRI and IRI samples (Newman et al., 2015). Spearman’s correlation analysis was further used to evaluate associations between CRDEG expression and the relative abundance of 22 immune cell types. To further explore the biological implications of the identified CRDEGs, the expression levels of representative inflammation-, apoptosis-, and proliferation-related factors were compared between non-IRI and IRI samples. Spearman correlation analysis was performed to assess the associations between CRDEG expression and these factors.

### 2.7 Clustering and cluster analysis of CRDEGs

To explore CRDEG-related molecular heterogeneity among KIRI samples, consensus clustering was performed on 203 KIRI samples according to the expression profiles of the four CRDEGs using the R package “ConsensusclusterPlus” (Wilkerson and Hayes, 2010). Consensus clustering was performed with a maximum cluster number of 9 and 50 resampling iterations. For each iteration, 80% of the samples and 100% of the features were randomly selected. K-means clustering was applied using Euclidean distance measurements, with the random seed set to 123456. Then, the expression of the four CRDEGs in the KIRI subgroups was calculated and DEGs between the CRDEG-based subgroups were identified. Finally, KEGG enrichment analysis of DEGs was performed using the “clusterProfiler” software package in R.

### 2.8 Acquisition and processing of single-cell RNA sequencing data

The murine scRNA-seq dataset (GSE161201) comprises a control kidney sample and two ischemia ‒reperfusion injury (IRI) samples collected at 6 h and 24 h post-reperfusion. Single-cell data were processed using the “Seurat” (Butler et al., 2018) and “Harmony” (Korsunsky et al., 2019) packages in R. Cells were retained according to the following quality control criteria: 500–3000 detected genes per cell, mitochondrial gene percentage <20%, and ribosomal gene percentage <10%. After normalization and identification of highly variable genes, batch effects among samples were corrected using Harmony. Dimensionality reduction, clustering, and visualization were performed using tSNE/UMAP. Cluster-defining genes were determined through Seurat’s “FindAllMarkers” feature. Genes meeting selection thresholds were required to be expressed in a minimum of 25% of target population cells while having a minimum log_2_FC exceeding 0.25. For each cellular cluster, odds ratios (ORs) were computed, followed by characterization of metacell distributions across tissues or specimens (Zheng et al., 2021). To directly evaluate the distribution of CRDEG expression across annotated cell clusters and samples, violin plots and UMAP-based feature plots were generated for *PPP1R15A, LIPT1, SERPINE1,* and *HSPD1*. Intercellular communication patterns among annotated cell types were inferred using the “CellChat” R package (www.cellchat.org). (Jin et al., 2021) The global interaction number and interaction strength were visualized, and pathway-level communication patterns were further analyzed using incoming and outgoing signaling role heatmaps. Among the CellChat-inferred signaling pathways, SPP1-, VEGF-, and TNF-related pathways were selected as representative pathways for ligand–receptor interaction visualization based on their biological relevance to injury repair, vascular response, inflammatory activation, and immune-cell recruitment in KIRI.

### 2.9 Animal experimental validation

Twenty-two 8-week-old male C57BL/6 mice were obtained from Speifu (Beijing, China). All the animals were maintained under pathogen-free conditions with a 12 h/12 h light/dark cycle, provided unrestricted access to food and water, and allowed 7 days for environmental adaptation prior to experimentation. The mice were divided randomly into four experimental groups: sham-operated group, KIRI group, KIRI + ATTM (ammonium tetrathiomolybdate, Macklin, A828261) group, and KIRI + ES-Cu (elesclomol, MCE, 488832-69-5 plus CuCl_2_, Macklin, C804817) group (N = 6 in the first three groups and N = 4 in the last group). KIRI was induced using the following protocol: First, anesthesia was induced via intraperitoneal injection of a stable, commercially available, premixed 1.25% (w/v) tribromoethanol solution (Aibei Biotechnology, Nanjing, China) at a dose of 200 mg/kg. This dose and formulation were selected to ensure a surgical plane of anesthesia (as evidenced by the loss of pedal and palpebral reflexes) while mitigating the risks associated with the decomposition of self-prepared solutions. The depth of anesthesia was monitored throughout the procedure. Body temperature was maintained at 34 °C–36 °C using a thermostatically controlled heating pad to prevent anesthesia-induced hypothermia. Next, the bilateral kidney hila were clamped with nondestructive arterial clips for 30 min at a temperature of 34 °C–36 °C. Following this, reperfusion was allowed for 24 h, after which the mice were evaluated through pathology and biochemical assays. In the sham operation group laparotomy but not bilateral kidney hilum clamping was performed. ATTM administration: 10 mg/kg ATTM was administered intraperitoneally to mice daily for 7 days, after which they underwent the same surgery as the KIRI group on day 8 (Song et al., 2011; Qi et al., 2023). The ES-Cu treatment regimen consisted of daily intraperitoneal delivery of 3 mg/kg elesclomol combined with 0.5 mg/kg copper chloride to the experimental animals, followed by the same procedure performed on day 13 as that performed in the KIRI group (Cai et al., 2025). Twenty-four hours after the surgery, blood samples and bilateral kidney tissues were collected from all the mice. Following sample collection, the mice were euthanized by cervical dislocation under deep anesthesia, and death was confirmed by the cessation of respiration and heartbeat. Euthanasia was performed in accordance with the American Veterinary Medical Association (AVMA) Guidelines for the Euthanasia of Animals (Underwood and Anthony, 2020). All animal procedures were approved by the Medical Ethics Committee of the Second Affiliated Hospital of Guangxi Medical University [No. 2025-KYL (016)] and were conducted in accordance with institutional and national guidelines for the care and use of laboratory animals. The study was also reported in accordance with the Animal Research: Reporting of *In Vivo* Experiments (ARRIVE) guidelines 2.0 (Percie du Sert et al., 2020).

### 2.10 Kidney injury assessment and quantitative real-time PCR/ RT‒qPCR

Blood was drawn from the ocular venous plexus of the study animals and promptly preserved in sterilized vials maintained at 4 °C. Serum creatinine and blood urea nitrogen concentrations were quantified using a clinical chemistry automated analyzer. We performed H&E staining on kidney tissue to evaluate kidney injury. Renal copper ion levels were measured using a copper assay kit according to the manufacturer’s instructions, and apoptosis in kidney tissues was assessed by TUNEL staining (Cai et al., 2022). Finally, we extracted total RNA from kidney samples and assessed the relative mRNA expression levels of CRDEGs. The nucleotide sequences of all the primers employed in this study are provided in Table 1.

**TABLE 1:** Nucleotide sequences of primers used for qRT-PCR.

Gene symbol	Forward primer (5′->3′)	Reverse primer (5′->3′)
Ppp1r15a	TAACAGGAGCAGATCAGATAGAAGC	AGGCTTCTCTCTAGAGTAGTACTCC
Lipt1	TCATCGGCAGGCATCAGAATC	CATGTCGTGGTACACGGCTC
Serpine1	TCTGGGAAAGGGTTCACTTTACC	GACACGCCATAGGGAGAGAAG
Hspd1	GCCTTAATGCTTCAAGGTGTAGA	CCCCATCTTTTGTTACTTTGGGA
Gapdh	TGCACCACCAACTGCTTAGC	GGATGCAGGGATGATGTTCT

### 2.11 Molecular network prediction

To explore potential regulatory relationships involving the identified CRDEGs, we created networks for microRNA (miRNA) –mRNA and transcription factors (TF) –mRNA interactions. Candidate miRNAs targeting the CRDEGs were retrieved from the miRTarBase v9.0 and TarBase v9.0 databases (https://mirtarbase.cuhk.edu.cn and https://dianalab.e-ce.uth.gr/tarbasev9), followed by network visualization of miRNA–mRNA interactions through Cytoscape version 3.9.1. Next, we utilized the TRRUST and ChEA3 databases (https://www.networkanalyst.ca/) to predict the transcription factors associated with the biomarkers. Subsequently, we created a TF–mRNA network (Peng et al., 2021; Shen et al., 2022).

### 2.12 Analysis of drug–gene interactions

The DGIdb platform (www.dgidb.org) serves as a comprehensive web-based repository containing curated data on gene–drug interactions and potentially targetable genes, compiled from multiple scientific sources and databases (Freshour et al., 2021). A total of potential candidate drugs interacting with CRDEGs were identified through this resource.

### 2.13 Statistical analysis

The results are expressed as the mean values ± standard deviations (SDs). Statistical analyses were conducted using the R software. Intergroup comparisons were evaluated using either the Wilcoxon rank-sum test or Student’s t test with a significance threshold set at p < 0.05.

## 3 Results

### 3.1 Identification of CRDEGs

**FIGURE 1:**
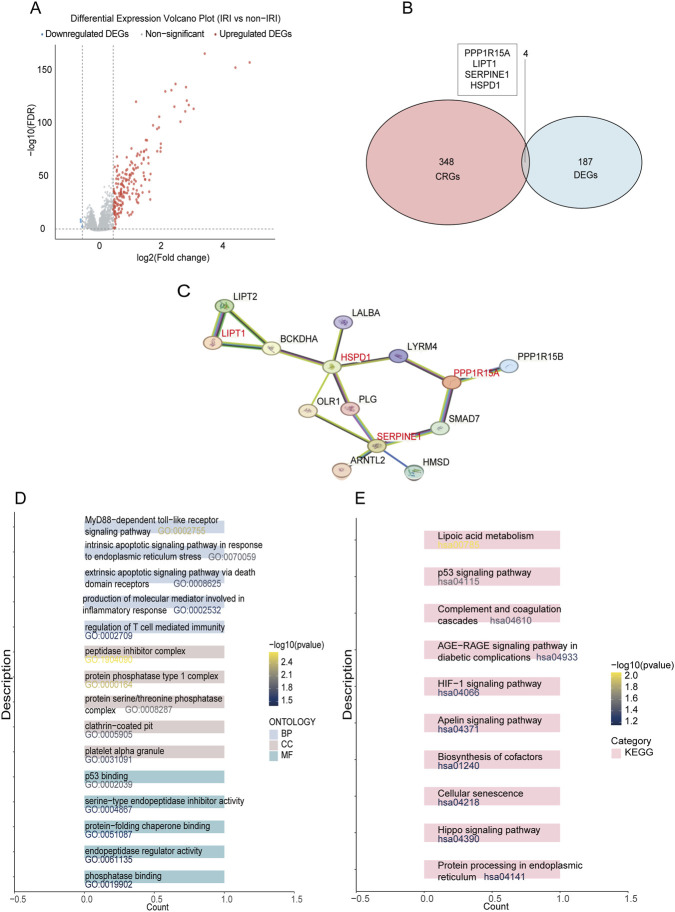
Identification and Functional Characterization of CRDEGs. **(A)** Volcano plot of DEGs between IRI and non-IRI samples. **(B)** Intersection between DEGs and CRGs. **(C)** PPI network of the four CRDEGs and their interacting proteins. **(D)** GO enrichment analysis of CRDEGs including biological processes, cellular components, and molecular function terms. **(E)** KEGG pathway analysis of CRDEGs.

In the GSE43974 dataset, we identified 191 DEGs, including three downregulated genes and 188 upregulated genes (Figure 1A). By intersecting these 191 DEGs with the 352 CRGs, we obtained four candidate CRDEGs, namely, *PPP1R15A, LIPT1, SERPINE1,* and *HSPD1* (Figure 1B).

### 3.2 CRDEGs interactions and functional enrichment

To explore the potential biological relevance of these CRDEGs in KIRI, a protein ‒protein interaction (PPI) network was constructed. The PPI network indicated interactions between the four CRDEGs and several related proteins, suggesting potential functional connections among these genes and their interacting partners (Figure 1C). Further functional enrichment analyses revealed that the CRDEGs are mainly involved in Toll ‒like receptor ‒mediated immune ‒inflammatory responses, endoplasmic reticulum stress ‒triggered and death receptor ‒triggered apoptosis, and T-cell immunomodulation. In addition, KEGG pathway analysis suggested enrichment in several disease- and stress-related pathways, including lipoic acid metabolism, p53 signaling, complement and coagulation cascades, HIF-1 signaling and protein processing in the endoplasmic reticulum (Figures 1D,E).

### 3.3 Diagnostic value of the four CRDEGs in KIRI

**FIGURE 2:**
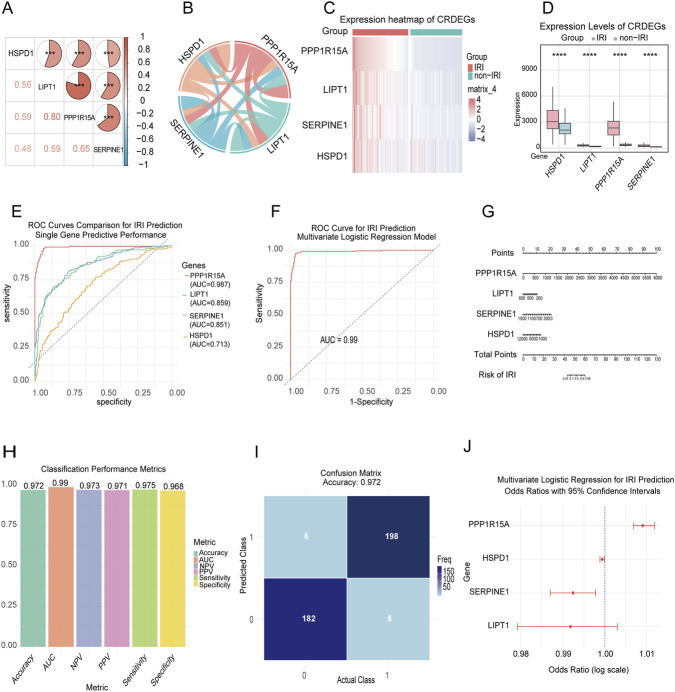
Diagnostic value of 4 CRDEGs in KIRI. **(A)** Heatmap of correlations among the four CRDEGs. **(B)** Chord diagram illustrating the associations among the four CRDEGs. **(C)** Heatmaps of the expression levels of the four CRDEGs between IRI and non-IRI. **(D)** Box plots showing the expression levels of the four CRDEGs between IRI and non-IRI samples. **(E)** ROC Analysis of Single-Gene Predictors for IRI. **(F)** ROC Curve of the Multivariable Model for Predicting IRI. **(G)** Nomogram model for predicting IRI risk based on the four CRDEGs. **(H)** Performance Metrics of the Multivariable Model for IRI Prediction. **(I)** Confusion Matrix Heatmap of the Multivariable Model for IRI Prediction. **(J)** Forest plot of multivariate logistic regression analysis for IRI prediction. Statistical significance is indicated as ***P < 0.001 and ****P < 0.0001.

We analyzed the relationships between four CRDEGs, *PPP1R15A, LIPT1, SERPINE1,* and *HSPD1*, and performed correlation analyses in the discovery cohort. The results revealed that the four CRDEGs were positively correlated with each other, with the strongest correlation observed between *LIPT1* and *PPP1R15A* (r = 0.80). The correlation coefficients between *HSPD1* and *PPP1R15A, LIPT1 and SERPINE1*, and *HSPD1* and *SERPINE1* were 0.59, 0.59, and 0.48, respectively (Figures 2A,B). The grouped expression heatmaps (Figure 2C) and box plots (Figure 2D) of the four CRDEGs showed that all the genes were expressed at significantly higher levels in the IRI samples than in the non-IRI samples, with *PPP1R15A* showing the most pronounced between-group difference. Single-gene ROC curve analysis was performed to assess the diagnostic value of each CRDEG for KIRI. *PPP1R15A* had the highest AUC value (0.987), whereas *LIPT1*, *SERPINE1,* and *HSPD1* showed AUC values of 0.859, 0.851, and 0.713, respectively, indicating varying degrees of discriminatory ability (Figure 2E). To further evaluate the combined diagnostic performance of these genes, we constructed a multivariate logistic regression model and column–line plots based on the four CRDEGs. Model performance was then assessed using ROC curves and confusion matrices. In the discovery cohort, the multivariate model showed high apparent discriminative performance, with an AUC of 0.99 (Figures 2F,G). The model achieved an accuracy of 97.2%; a sensitivity of 97.5%; a specificity of 96.8%; and positive and negative predictive values of 97.1% and 97.3%, respectively (Figures 2H,I). Multivariate logistic regression analysis suggested that *PPP1R15A* was positively associated with IRI status (OR = 1.01; 95% CI: 1.01–1.01; P = 6.14 × 10^−13^), whereas *SERPINE1* (OR = 0.99; P = 7.12 × 10^−3^) was inversely associated with IRI status (Figure 2J). Together, these findings suggest that the four CRDEGs have potential diagnostic relevance for distinguishing IRI from non-IRI samples, although further external validation is needed to confirm the generalizability of the model.

### 3.4 Enrichment analysis for each of the four CRDEGs

**FIGURE 3:**
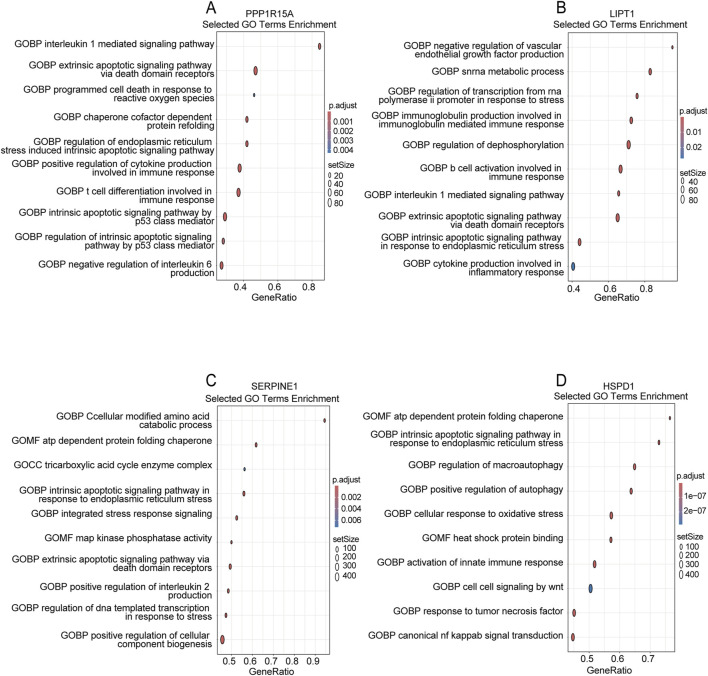
Single-gene GSEA of the four CRDEGs. GSEA results for PPP1R15A **(A)**, LIPT1 **(B)**, SERPINE1 **(C)**, and HSPD1 **(D)** in biological pathways.

A genewise GSEA was conducted to explore the potential mechanisms underlying the involvement of the four CRDEGs in KIRI (Figure 3). Interestingly, *PPP1R15A*-related enrichment was associated mainly with interleukin-1-mediated signaling, exogenous apoptosis (death domain receptor-mediated), reactive oxygen species-responsive programmed cell death, immune-associated T-cell differentiation, and positive regulation of cytokine production. *LIPT1* was enriched in inflammatory and immune-related processes, including interleukin-1-mediated signaling, B-cell activation, immunoglobulin production, and endogenous/exogenous apoptotic pathways. *SERPINE1* related enrichment was linked to integration of stress response signaling, endogenous/exogenous apoptosis, positive regulation of interleukin two production, and DNA transcriptional regulation in response to stress. *HSPD1* was predominantly associated with ATP-dependent protein folding and heat shock protein binding and is also associated with endoplasmic reticulum stress apoptosis, the oxidative stress response, NF-κB signaling, and innate immune activation. Taken together, these enrichment patterns suggest that the four CRDEGs are associated with immune activation, cellular stress response, and regulated cell death-related processes in KIRI. These results provide hypothesis-generating clues regarding possible biological connections between cuproptosis-related transcriptional changes and KIRI-associated injury responses.

### 3.5 Immune cell infiltration and cell death analysis of the four CRDEGs in KIRI

**FIGURE 4:**
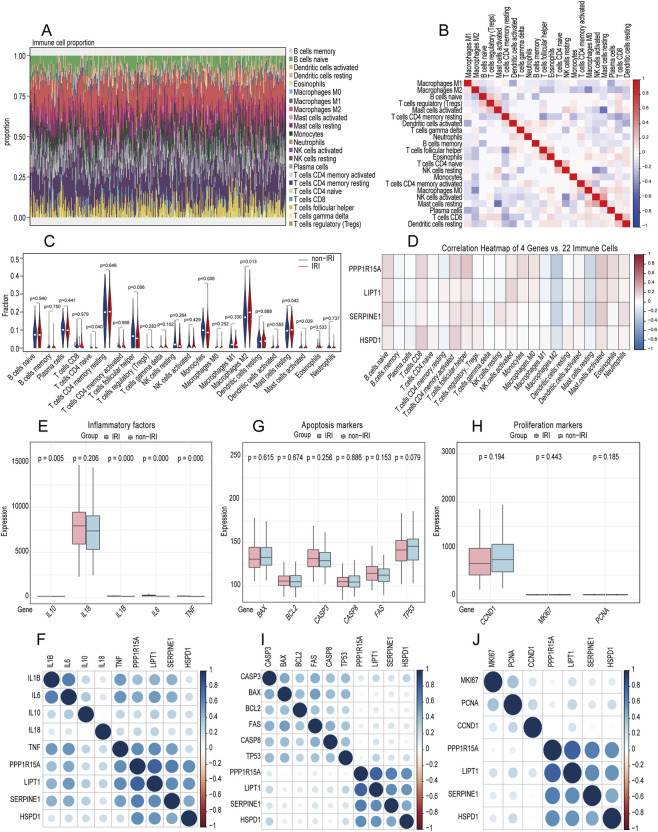
Immune infiltration and cell death analysis of four CRDEGs. **(A)** Stacked bar plot showing the relative proportions of 22 immune cell types in each sample. **(B)** Correlation analysis of immune cells in the KIRI group. **(C)** Violin plots of immune cell infiltration differences between IRI and non-IRI groups. **(D)** Correlation analysis of four CRDEGs and immune cells. **(E)** Comparison of inflammatory factors between the IRI and non-IRI groups. **(F)** Correlation analysis between four CRDEGs and inflammatory factors. **(G)** Comparison of apoptosis factors between the IRI and non-IRI groups. **(H)** Comparison of cell proliferation factors between the IRI and non-IRI groups. **(I)** Correlation analysis between four CRDEGs and apoptosis-related markers. **(J)** Correlation analysis between four CRDEGs and proliferation-related markers.

Functional annotation and single-gene GSEA suggested that immunological reactions, apoptosis, oxidative stress, and inflammatory processes may be involved in KIRI. To further investigate the potential biological role of these four CRDEGs in KIRI, we performed immune infiltration and cell death-related analyses. The CIBERSORT algorithm was used to estimate the relative proportions of 22 immune cell types in each sample, and the overall immune infiltration is presented in Figure 4A. Additionally, we performed an immune cell correlation analysis within the KIRI group, which revealed that the strongest positive correlation was between activated NK cells and resting mast cells (Figure 4B). Comparative immune profiling identified six cell populations whose abundance significantly differed between the IRI and non-IRI groups: naive CD4^+^ T cells, follicular helper T cells, monocytes, M2 macrophages, activated mast cells and resting mast cells (Figure 4C). Correlation analysis further revealed that *HSPD1* expression was positively associated with activated cytotoxic T-cell infiltration. In contrast, *PPP1R15A, LIPT1*, and *SERPINE1* expression was negatively correlated with M2-type macrophages and resting mast cells, and positively correlated with eosinophils and activated mast cells (Figure 4D).

Given the observed alterations in immune cell infiltration, especially macrophage-related changes, we further evaluated the expression levels of the cytokines interleukin 1 B (IL1B), interleukin-6 (IL-6), interleukin-10 (IL-10), interleukin-18 (IL-18), and tumor necrosis factor (TNF). As shown in Figure 4E, the levels of IL1B, IL-6, IL-10, IL-18 and TNF were higher in the IRI group than in the non-IRI group. Further analysis revealed that the genes co-regulated of these four CRDEGs appeared to be positively correlated with IL1B, IL-6, IL-10, IL-18, and TNF, and these results suggest that immune response plays a role in KIRI (Figure 4F).

Next, we evaluated apoptosis- and proliferation-related gene expressions in the IRI and non-IRI groups. We selected CASP3, BAX, BCL2, FAS, CASP8, and TP53 as indicators of apoptosis, and MKI67, PCNA, and CCND1 as indicators of cell proliferation. Although no statistically significant differences were observed for these selected markers between the two groups, correlation analyses indicated that the four CRDEGs were positively correlated with several apoptosis- and proliferation-related genes (Figures 4G–J). These findings suggest that the four CRDEGs are associated with immune infiltration, inflammatory responses, and cell-death-related biological processes in KIRI, but do not establish direct regulatory relationships.

### 3.6 Clustering and cluster analysis of KIRI

**FIGURE 5:**
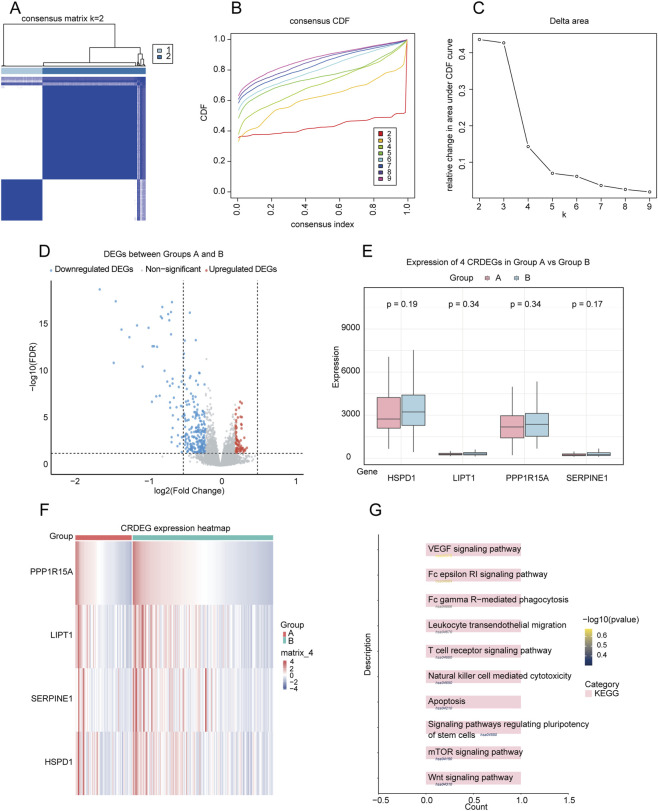
CRDEG-based clustering and subtype analysis of KIRI. **(A)** Consensus clustering heatmap with consensus matrix (k = 2). **(B)** Consensus Cumulative Distribution Function (CDF) Curve. **(C)** Delta area curve for determining the optimal k value. **(D)** Volcano plot of differentially expressed genes between Groups A and B. **(E)** Expression levels of the four CRDEGs in Group A and Group B. **(F)** Heatmap showing the expression patterns of the four CRDEGs in Groups A and B. **(G)** KEGG enrichment analysis of DEGs between Groups A and B.

To investigate whether the four CRDEGs could further stratify KIRI samples into molecularly distinct subtypes, we performed consensus clustering based on the expression profiles of *PPP1R15A, LIPT1, SERPINE1,* and *HSPD1*. A total of 203 KIRI samples were classified into two subgroups (Group A, n = 58; Group B, n = 145) according to the optimal clustering pattern, which was determined by the consensus matrix, cumulative distribution function (CDF) curve, and delta area analysis (Figures 5A–C). This subtype analysis was conducted to determine whether the expression heterogeneity of CRDEGs reflects distinct biological states within KIRI rather than merely distinguishing KIRI from non-IRI samples. Differential expression analysis of the samples from the two groups identified 77 DEGs (Figure 5D). We further compared the expression levels of the four CRDEGs between Subtype A and Subtype B. Although slight differences were observed, none of the four CRDEGs—*PPP1R15A*, *LIPT1*, *SERPINE1*, and *HSPD1*—significantly differed between the two subtypes (Figures 5E,F). Subsequently, we performed KEGG enrichment analysis to characterize the biological features associated with these subtypes. The results of this analysis showed that the DEGs were enriched mainly in immune-related pathways, including leukocyte migration, T-cell- and NK-cell-associated immune responses, apoptosis and VEGF-, mTOR-, and Wnt-related signaling pathways (Figure 5G). These findings suggest that CRDEG-based stratification may be associated with distinct immune–inflammatory and cell-death-related characteristics within KIRI.

### 3.7 External validation of the four CRDEGs diagnostic model

**FIGURE 6:**
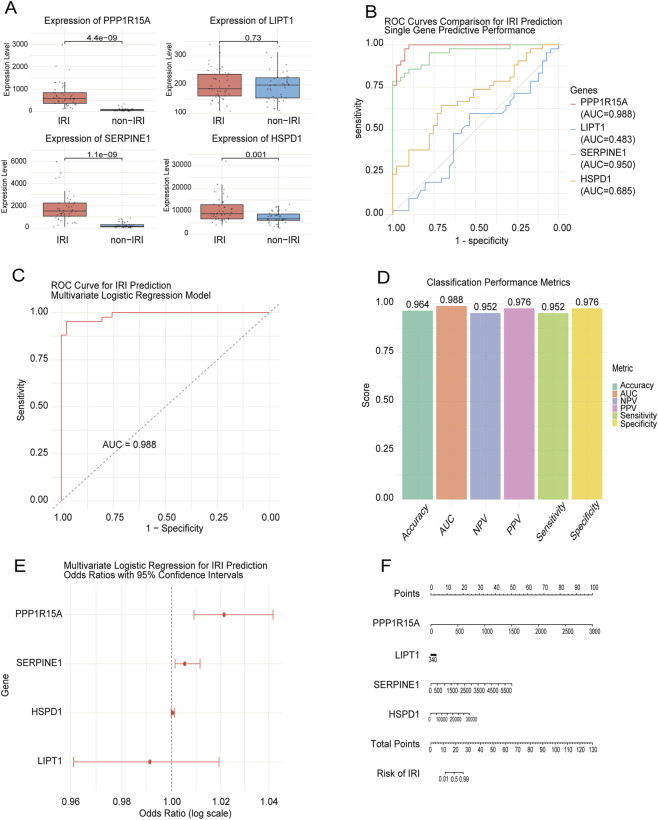
External validation of the four-CRDEG diagnostic model in the GSE126805 dataset. **(A)** Expression levels of four CRDEGs between IRI and non-IRI in the GSE126805 dataset. **(B)** ROC curves of Single-Gene Predictors for IRI diagnosis. **(C)** ROC Curve of the multivariable logistic regression model based on the four CRDEGs. **(D)** Classification performance metrics of the multivariate model, including accuracy, AUC, NPV, PPV, sensitivity, and specificity. **(E)** Forest plot of multivariate logistic regression analysis for IRI prediction. **(F)** Nomogram model for estimating IRI risk based on the four CRDEGs.

To further evaluate the robustness and generalizability of the diagnostic model based on the four CRDEGs, we performed external validation using the independent dataset GSE126805 (containing 83 samples, 42 in the IRI group and 41 in the non-IRI group). This dataset was excluded from model construction, thereby reducing the possibility of data leakage between the training and validation analyses. In the external validation dataset, the expression of *PPP1R15A, SERPINE1,* and *HSPD1* were upregulated in the IRI group compared with the non-IRI group, while *LIPT1* showed no significant difference between groups (P = 0.73, NS) (Figure 6A). In terms of single-gene prediction performance, *PPP1R15A* (AUC = 0.988) and *SERPINE1* (AUC = 0.950) showed excellent performance, and *HSPD1* showed moderate predictive performance with an AUC of 0.685, whereas *LIPT1* showed limited diagnostic value with an AUC of 0.483 (Figure 6B). The multivariate logistic regression model based on the four CRDEGs showed strong diagnostic performance on the GSE126805 dataset, with an AUC of 0.988, accuracy of 0.964, sensitivity of 0.952, and specificity of 0.976 (Figures 6C,D). Multivariate logistic regression analysis further suggested that *PPP1R15A* and *SERPINE1* were more strongly associated with IRI prediction than the other two CRDEGs in this validation cohort (Figure 6E). Meanwhile, the nomogram based on the four-CRDEGs model provided a visual tool for estimating the risk of IRI (Figure 6F). Collectively, these results provide validation support for the potential diagnostic relevance of the four-CRDEG model in distinguishing IRI samples from non-IRI samples in an independent public GEO cohort. Nevertheless, because both the discovery and validation datasets were derived from public transcriptomic repositories and the AUC values were relatively high, these findings should be interpreted cautiously. Further validation in larger, prospectively collected, multi-center clinical cohorts is required to determine the true generalizability and clinical utility of the model.

### 3.8 Validation using the murine single-cell transcriptomic data

**FIGURE 7:**
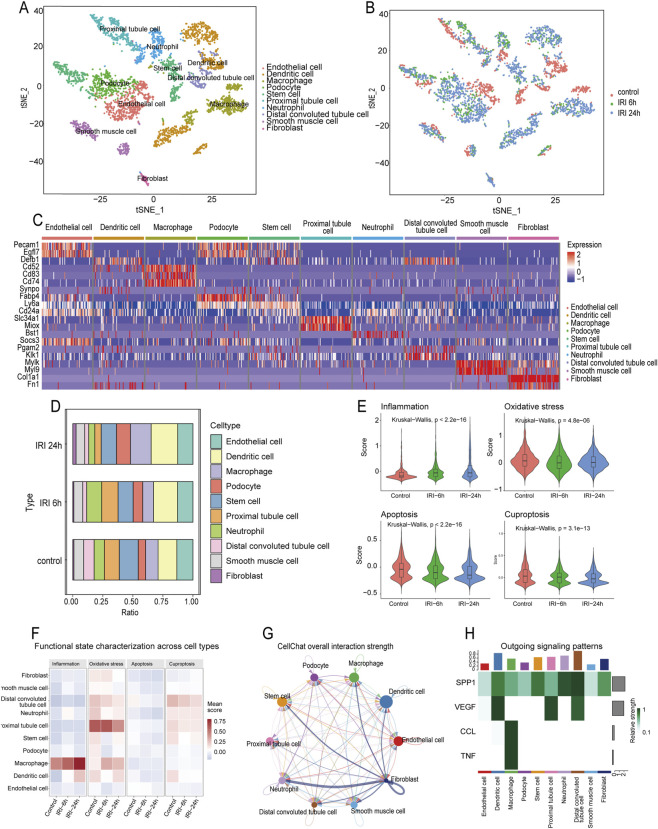
Single-cell transcriptomic validation and functional characterization in KIRI. **(A)** t-SNE plot showing the annotation of ten major cell populations in the murine single-cell RNA-seq dataset. **(B)** t-SNE plot showing the distribution of cells from the control, IRI-6h, and IRI-24 h groups. **(C)** Heatmap showing the expression patterns of representative marker genes across the ten annotated cell types. **(D)** Stacked bar plot showing the relative proportions of the ten cell types across the control, IRI-6h, and IRI-24 h groups. **(E)** Violin plots showing the differences in functional state scores, including inflammation, oxidative stress, apoptosis, and cuproptosis, among the control, IRI-6h, and IRI-24 h groups. Statistical significance was assessed using the Kruskal–Wallis test. **(F)** Heatmap showing the mean functional state scores of inflammation, oxidative stress, apoptosis, and cuproptosis across different cell types and experimental groups. **(G)** CellChat-based cell–cell communication network showing the overall interaction strength among the ten annotated cell types. **(H)** Heatmap showing outgoing signaling patterns of key pathways identified by CellChat analysis, including SPP1, VEGF, CCL, and TNF signaling.

The murine single-cell RNA-seq dataset GSE161201 was used to further evaluate the cellular distribution, functional state changes, and intercellular communication patterns associated with the four CRDEGs in KIRI. The dataset included 22,310 cells distributed across three experimental conditions: 5,246 cells from control samples (GSM4891840), 10,393 cells from 6 h post-reperfusion samples (GSM4891841), and 6,671 cells from 24 h post-reperfusion samples (GSM4891842). After quality control, dimensionality reduction, and batch effect correction, 3,786 high-quality cells were retained, including 1,052 from the control sample, 892 from the IRI-6 h sample, and 1,842 from the IRI-24 h sample. As shown in Figures 7A–C, ten distinct cellular populations were identified through manual cell type annotation using canonical marker genes from published literature and established cellular annotation databases. These cell types included stem cells, dendritic cells, distal tubule cells, neutrophils, proximal tubule cells, endothelial cells, macrophages, smooth muscle cells, podocytes, and fibroblasts. Cell proportion analysis revealed dynamic changes in cellular composition after IRI, with increased dendritic cell abundance in the IRI-24 h group and reduced proportions of several renal tubular cell populations, including proximal and distal convoluted tubule cells (Figure 7D; Supplementary Figure 2A).

To characterize injury-associated transcriptional states, we calculated module scores for inflammation, oxidative stress, apoptosis, and cuproptosis. Compared with the control group, the IRI-6 h and IRI-24 h groups exhibited significant alterations in these functional state scores, indicating the activation of inflammation-, stress-, and cell-death-related transcriptional programs during KIRI progression (Figure 7E; Supplementary Figures 2B,C). Cell-type-level analysis further revealed that these functional states were heterogeneously distributed across renal cell populations: inflammation-related scores were relatively high in macrophages, oxidative stress-related scores were prominent in proximal tubule cells, while apoptosis- and cuproptosis-related scores were elevated in distal convoluted tubule cells (Figure 7F). Direct visualization of the four CRDEGs across annotated cell populations showed heterogeneous expression patterns. *Ppp1r15a* and *Hspd1* were broadly expressed in multiple renal cell populations, while *Lipt1* and *Serpine1* exhibited weak and sparse expression (Supplementary Figure 1).

CellChat analysis revealed extensive intercellular communication among immune, endothelial, stromal, and renal parenchymal cells (Figure 7G; Supplementary Figures 2D–E). Representative signaling pathways, including SPP1, VEGF, and TNF, were involved in the inferred communication network, with fibroblasts, endothelial cells, macrophages, dendritic cells, and neutrophils showing relatively active signaling roles (Figure 7H; Supplementary Figure 3). These findings suggest that KIRI is associated with altered cell composition, functional state remodeling, heterogeneous CRDEG expression, and changes in intercellular communication.

### 3.9 Experimental validation of cuproptosis-related injury and candidate CRDEG expression in murine KIRI

**FIGURE 8:**
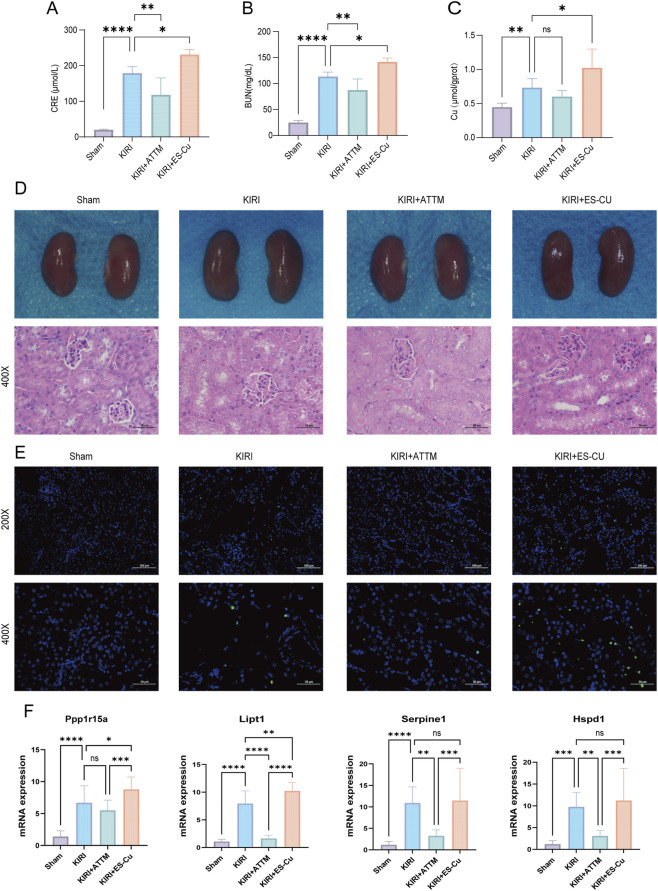
Effects of cuproptosis modulation on renal injury and CRDEG expression in murine KIRI. **(A,B)** Serum CRE and BUN levels in the Sham, KIRI, KIRI + ATTM, and KIRI + ES-Cu groups. **(C)** Renal copper content in different groups. **(D)** Representative gross kidney images and H&E staining of kidney tissues. **(E)** TUNEL staining of kidney tissues. **(F)** Relative mRNA expression levels of four CRDEGs in different groups. Scale bars are shown for microscopic images. n = 6 in the Sham, KIRI, and KIRI + ATTM groups; n = 4 in the KIRI + ES-Cu group. Statistical significance is indicated as *P < 0.05, **P < 0.01, ***P < 0.001, ****P < 0.0001; ns, not significant.

To further evaluate cuproptosis-related injury responses in KIRI, we established a murine KIRI model and treated it with the cuproptosis inhibitor ATTM or the cuproptosis inducer ES-Cu. Compared with the Sham group, the KIRI group showed significantly increased blood urea nitrogen (BUN) and creatinine (CRE) levels, indicating impaired renal function. ATTM treatment partially alleviated renal dysfunction, whereas ES-Cu treatment further exacerbated renal injury (Figures 8A,B). Renal copper content was also increased after KIRI and was further elevated in the ES-Cu-treated group, while ATTM showed no significant reduction compared with the KIRI group (Figure 8C). Representative gross kidney images and H&E staining revealed renal morphological and histopathological changes after KIRI, including tubular epithelial damage, tubular dilation, vacuolization, and cast formation. These pathological changes appeared to be less pronounced in the KIRI + ATTM group and more evident in the KIRI + ES-Cu group (Figure 8D). TUNEL staining showed increased TUNEL-positive signals after KIRI, which appeared to be reduced by ATTM treatment and increased by ES-Cu treatment (Figure 8E). Whole-kidney qPCR analysis further showed that *Ppp1r15a, Lipt1, Serpine1,* and *Hspd1* were upregulated in KIRI mice compared with Sham controls. ATTM treatment reduced the expression of these CRDEGs, particularly *Lipt1, Serpine1,* and *Hspd1*, whereas ES-Cu treatment further increased *Ppp1r15a* and *Lipt1* expression (Figure 8F). Together, these findings provide preliminary tissue-level support for an association between cuproptosis-related injury responses and altered CRDEG expression in murine KIRI, but do not establish direct functional causality.

### 3.10 Prediction of upstream regulators and drug ‒gene interactions for the four CRDEGs

**FIGURE 9:**
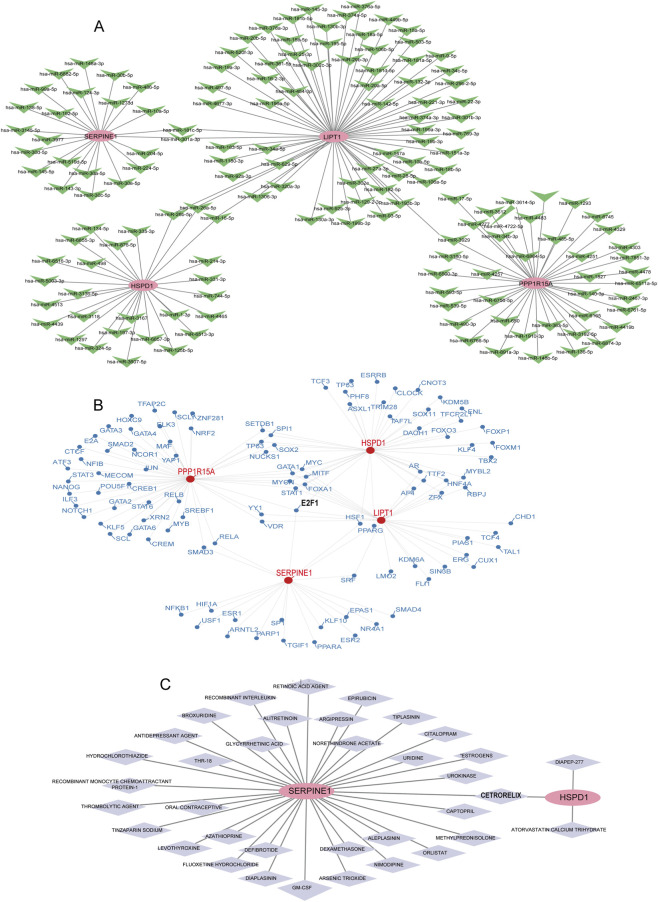
Predicted regulatory and drug-gene interaction networks of the four CRDEGs. **(A)** Predicted miRNA-CRDEG interaction network. **(B)** Predicted TF-CRDEG interaction network. **(C)** Predicted drug-gene interaction network. Red nodes represent CRDEGs, and surrounding nodes represent predicted miRNAs, transcription factors, or candidate compounds.

To further explore the potential upstream regulators and therapeutic relevance of the four CRDEGs, we constructed TF *‒*CRDEG, miRNA *‒*CRDEG and drug *‒*gene interaction networks. The miRNA *‒*CRDEG network identified 152 candidate miRNAs, and TF *‒*CRDEGs network revealed 103 candidate transcription factors potentially associated with the four CRDEGs (Figures 9A,B). Among these candidates, E2F1 was connected with multiple CRDEGs and appeared as a central node in the network. Drug *‒*gene interaction prediction identified 36 candidate compounds, mainly for interactions with *SERPINE1* and *HSPD1*. CETRORELIX was predicted to interact with both genes (Figure 9C). These predicted regulatory and drug*‒*gene interaction networks provide candidate targets for future mechanistic and therapeutic studies but require further experimental validation.

## 4 Discussion

The kidney is a highly perfused and metabolically active organ and is therefore highly vulnerable to IRI. KIRI commonly occurs during renal surgery, kidney transplantation, extracorporeal shockwave lithotripsy, and other ischemic renal conditions and is an important contributor to acute renal injury, delayed graft function and transplant rejection (Micó-Carnero et al., 2022; Knijff et al., 2022). During kidney ischemia, inadequate oxygen supply and metabolite accumulation induce mitochondrial dysfunction and cellular injury. Although reperfusion restores blood flow, it can further exacerbate renal damage through calcium overload, triggering oxidative stress, inflammatory activation, and regulated cell death (Quader et al., 2021; Granata et al., 2022). These processes indicate that KIRI is not caused by a single pathological event but involves an interaction among metabolic stress, the immune response, and cell death pathways (Pefanis et al., 2019; Sanz et al., 2023). Cuproptosis was first described by Tsvetkov et al., in 2022 as a copper-dependent form of regulated cell death (Tsvetkov et al., 2022). This process occurs when copper overload disrupts mitochondrial protein homeostasis by interacting with lipoylated TCA cycle enzymes, which results in abnormal aggregation of lipoylated proteins, depletion of iron–sulfur cluster proteins, proteotoxic stress, mitochondrial dysfunction, and eventual cell death (Tsvetkov et al., 2022). Because mitochondrial dysfunction, oxidative stress, and regulated cell death are central features of KIRI, cuproptosis-related genes may provide a potential link between metabolic injury and downstream inflammatory or apoptotic responses. Recent evidence has also implicated copper homeostasis in IRI, suggesting that copper-dependent stress responses may have broader relevance in ischemic tissue damage (Guo et al., 2023). Therefore, exploring cuproptosis-related genes in KIRI may help identify candidate molecular mediators linked to metabolic stress, inflammation, and regulated cell death.

In this study, transcriptomic analysis and machine learning approaches were used to identify candidate cuproptosis-related genes associated with KIRI. Differential expression analysis of the GSE43974 dataset identified 191 DEGs between control and KIRI samples. Functional annotation revealed that these genes were mainly involved in regulation of inflammatory responses, reactive oxygen species (ROS) reactions, apoptosis, lymphocyte differentiation and cytokine or chemokine activity and were enriched in pathways such as the p53 signaling pathway, complement and coagulation cascade, and HIF-1 signaling pathway. These findings are consistent with previous evidence that oxidative stress, inflammation, and apoptosis are central pathological features of KIRI (Pefanis et al., 2019; Sanz et al., 2023; Ye et al., 2025).

By intersecting DEGs with cuproptosis-related genes and further screening them using machine-learning algorithms, four CRDEGs, *PPP1R15A, LIPT1, SERPINE1* and *HSPD1* were identified as candidate genes associated with KIRI. The expression levels of the four genes were significantly higher in the IRI samples than in the non-IRI samples, which suggests that these genes may participate in the renal stress response after IRI. Correlation and protein‒protein interaction analyses further indicated potential functional associations among these genes but did not confirm direct regulatory or synergistic relationships. The four-gene diagnostic model showed strong predictive performance, with *PPP1R15A* identified as an independent risk factor in the logistic regression model; however, given the high AUC values and limited external validation, its predictive value still requires confirmation in larger independent cohorts.

*PPP1R15A* is a stress-inducible gene involved in eIF2α dephosphorylation and recovery of protein synthesis after cellular stress (Zhang et al., 2024; Dalet et al., 2017). This function suggests that PPP1R15A may reflect integrated stress-response activation in KIRI rather than serving as direct evidence of cuproptosis regulation. *LIPT1* participates in TCA-cycle-dependent energy production and mitochondrial lipoic acid metabolism, providing the closest conceptual connection with canonical cuproptosis among the four CRDEGs, because cuproptosis depends on mitochondrial metabolism and lipoylated mitochondrial proteins (Tsvetkov et al., 2022; Liu J. et al., 2022). *SERPINE1* is involved in fibrinolysis, inflammation, tissue regeneration and kidney-injury-related senescence, suggesting that its upregulation may reflect inflammatory and repair-related remodeling during KIRI (Su et al., 2025; Liu R-j et al., 2022). *HSPD1* plays a versatile role in maintaining cellular proteome stability; sustaining mitochondrial function and energy metabolism; and regulating immune responses, including the induction of immune tolerance, and may therefore be related to mitochondrial injury and apoptosis-associated stress signaling (Aluksanasuwan et al., 2017). These gene-level annotations suggest that the four CRDEGs may reflect different components of KIRI-associated stress responses rather than a single linear regulatory pathway.

Consistently, single-gene GSEA and immune infiltration analyses showed that the four CRDEGs were associated with immune response, stress response and apoptosis. Significant changes in naive CD4^+^ T lymphocytes, follicular helper T cells, monocytic cells, M2 macrophages, and activated mast cells further support immune remodeling during KIRI. Together with the results of the apoptosis-related analysis, these findings suggest that immune activation and regulated cell death may cooperate in shaping renal injury after ischemia-reperfusion (Hasegawa et al., 2021). The immune-related associations may be partly explained by the complexity of disease- and cell-state-dependent immune gene-expression programs, as demonstrated in recent cross-disease analyses of CD4^+^ T-cell gene expression (Liao et al., 2025). Oxidative stress provides another plausible biological bridge, because redox imbalance can connect mitochondrial dysfunction, inflammatory activation, and regulated cell death during tissue injury (Liu et al., 2026). In addition, pan-cancer analyses of cuproptosis and copper metabolism-related gene sets have shown associations between copper metabolism-related genes, immune infiltration, pathway regulation, and drug sensitivity across disease contexts (Liu and Tang, 2022). These studies support the biological plausibility of our pathway-level findings, but they do not establish that p53 signaling, immune-related pathways, or apoptosis-related programs are functionally downstream of *PPP1R15A, LIPT1, SERPINE1,* or *HSPD1* in KIRI. Therefore, these findings should be interpreted as association-based evidence, and further functional studies are required to distinguish causal driver effects from secondary bystander changes.

Immune infiltration analysis suggested alterations in the immune landscape of KIRI. Macrophages are important regulators of inflammation and repair after AKI. In this study, we observed stable M1 macrophage levels but significantly elevated M2 populations in the KIRI group aligning with established findings (Wang et al., 2024). Previous work by Xie et al. (2022) demonstrated early kidney infiltration by M1 macrophages (≤48 h post-injury) followed by progressive M2 polarization in later stages. The increased M2 macrophage signature observed in our analysis may therefore reflect an injury-associated repair response, although this interpretation should be considered in the context of sampling time and the limitations of computational immune deconvolution. We found higher levels of IL1B, IL-6, IL-10, IL-18 and TNF in the IRI group than in the non-IRI group. These results indicate a robust immune response in KIRI, which is consistent with the results of prior research (Maryam et al., 2024). Correlations between the four CRDEGs and several immune cell subsets suggest that cuproptosis-related transcriptional changes may be associated with immune remodeling in KIRI.

KIRI involves multiple injury mechanisms including inflammatory reactions, apoptotic cell death, calcium overload, reactive oxygen species (ROS) production, protein kinase activation, endoplasmic reticulum stress, and mitochondrial dysfunction (Shao et al., 2021). KIRI triggers apoptosis in renal tubular epithelial cells, and excessive apoptosis leads to severe renal damage, which is a key driver of the progression of AKI (Zhang et al., 2022). In our analysis, selected apoptosis- and proliferation-related markers showed no significant differences in expression between IRI and non-IRI specimens. However, the four CRDEGs were positively correlated with multiple apoptosis- and proliferation-related genes. These findings imply a potential link between CRDEGs and cell death-associated transcriptional programs in KIRI, but do not confirm the direct regulation of apoptosis or proliferation.

Consensus clustering based on the four CRDEGs divided 203 KIRI samples into two molecular subgroups. Although the expression levels of *PPP1R15A, LIPT1, SERPINE1*, and *HSPD1* did not differ significantly between the two subtypes, differential expression analysis identified 77 genes whose expression patterns differed between Group A and Group B. KEGG enrichment analysis further suggested that these subtype-associated genes were mainly involved in immune cell proliferation and migration, apoptosis, the VEGF signaling pathway, the TGF-β signaling pathway, the mTOR signaling pathway and the Wnt signaling pathway. This grouping strategy has been validated in previous studies as an effective approach for identifying disease subtypes and uncovering core pathological mechanisms in renal diseases and KIRI (Xu et al., 2024). These findings suggest that CRDEG-based stratification may reflect immune–inflammatory and cell-death-related heterogeneity within KIRI, although the biological and clinical significance of these subgroups requires further validation.

External validation using GSE126805 dataset supported the diagnostic potential of the four-CRDEG model. In this cohort, *PPP1R15A* and *SERPINE1* showed relatively strong individual predictive performance, whereas *LIPT1* showed limited diagnostic value, suggesting that the contribution of each CRDEG may differ across datasets. Although GSE126805 was used as an independent public GEO validation cohort and was not involved in feature selection or model construction, this validation strategy cannot fully exclude dataset-specific bias. Both the discovery and validation datasets were obtained from public transcriptomic repositories and may be affected by shared technical and biological limitations, including sample composition bias, preprocessing differences, batch effects, tissue heterogeneity, and cell-mixture effects in bulk transcriptomic data. Previous studies have emphasized that gene expression analyses and bulk transcriptomic data mining are subject to correlation–causation ambiguity, cell-type composition effects, platform-related technical biases, and biological heterogeneity (Liu et al., 2024; Liu et al., 2025). Therefore, the high AUC values may partly reflect dataset-specific patterns rather than fully generalizable biological signatures. Larger, prospectively collected, multi-center clinical cohorts with standardized sample processing are needed to further evaluate the robustness and clinical applicability of the diagnostic model.

Single-cell analysis provided an exploratory cellular context for the bulk transcriptomic findings in KIRI. The altered proportions of immune and renal tubular cell populations were consistent with immune remodeling and tubular epithelial involvement during KIRI, which may partly explain why bulk transcriptomic analysis identified inflammatory, stress-response, and cell-death-related signals. Functional state scoring further showed cell type-dependent differences in scores related to inflammation, oxidative stress, apoptosis, and cuproptosis, suggesting that different renal cell populations may contribute differently to inflammatory activation, metabolic stress, and cell-death-related responses during KIRI. These findings are consistent with previous studies (Morris et al., 2022; Weng et al., 2021). Direct visualizations of the four CRDEGs further refined the interpretation of the bulk RNA-seq results. *Ppp1r15a* and *Hspd1* were broadly detectable across multiple cell populations, whereas *Lipt1* and *Serpine1* showed weak and sparse expression in this dataset. These findings suggest that expression changes observed in bulk kidney tissues may reflect both shifts in cell composition and gene expression changes within specific cell populations. CellChat further inferred potential communication among immune, endothelial, stromal, and renal parenchymal cells through representative SPP1-, VEGF-, and TNF-related signaling pathways. These pathways were selected for visualization based on their relevance to injury repair, vascular response, endothelial activation, and inflammatory signaling in KIRI. Together, these exploratory findings suggest that intercellular communication may be involved in inflammatory and repair-related responses within the injured kidney microenvironment, although validation in larger single-cell cohorts and targeted cell-type-specific experiments is still required.

In the murine KIRI model, KIRI was accompanied by impaired renal function, increased renal copper content, histopathological injury, and increased TUNEL-positive signals. ATTM treatment partially attenuated these injury-related changes, whereas ES-Cu treatment aggravated them. Whole-kidney qPCR further showed that *Ppp1r15a, Lipt1, Serpine1,* and *Hspd1* were upregulated after KIRI and were altered to varying degrees after ATTM or ES-Cu treatment. Together, these results provide experimental evidence that cuproptosis-related injury modulation is accompanied by changes in renal injury severity, apoptosis-related signals, and CRDEG expression in KIRI. Nevertheless, CRDEG-specific perturbation, rescue assays, and pathway-specific validation are still required to determine whether these genes directly regulate cuproptosis- or apoptosis-related signaling processes.

The predicted TF–CRDEG, miRNA–CRDEG, and drug–gene interaction networks provided exploratory information on potential upstream regulators and candidate compounds. These findings may provide preliminary clues for future KIRI intervention studies. This is consistent with the broader trend toward mechanism-based and precision treatment strategies, as discussed in cancer therapy research (Sonkin et al., 2024). However, because these analyses were based on public prediction databases, they should be regarded as hypothesis-generating and require experimental validation.

Overall, by combining bulk transcriptomic screening, external validation, immune-related characterization, exploratory single-cell analysis, tissue-level animal validation, and predicted TF–miRNA–drug interaction networks, this study provides a focused, multi-layered characterization of four candidate CRDEGs in KIRI. These complementary analyses helped connect *PPP1R15A, LIPT1, SERPINE1,* and *HSPD1* with tissue-level differential expression, diagnostic potential, cellular context, immune-inflammatory features, and putative regulatory or therapeutic associations. Nevertheless, these findings remain hypothesis-generating and should not be interpreted as evidence of a new computational framework or definitive causal mechanism. Future studies incorporating proteomic, metabolomic, epigenomic, spatial transcriptomic, and clinical outcome data with formal integrative modeling strategies are needed to achieve deeper cross-omics validation and to distinguish robust biological mechanisms from dataset- or analysis-specific associations (Yasmin et al., 2025). AI-driven integration of multi-omics and multimodal data may provide a useful framework for improving biomarker validation, model generalizability, and precision medicine-oriented interpretation in future KIRI studies (Liu, 2026).

This study has several limitations. Although external validation was performed, the available public datasets were limited, and the high AUC values should be interpreted cautiously because cohort heterogeneity, overfitting, and dataset-specific effects cannot be completely excluded. Future studies using larger independent cohorts and prospective samples are needed to further evaluate the generalizability of the diagnostic model. In addition, most transcriptomic analyses were based on bulk and cross-sectional data, which limits the ability to resolve cell-type-specific expression changes and temporal dynamics during the initiation, progression, and repair phases of KIRI. Although murine single-cell analysis and animal experiments provided additional biological context, cross-species differences and the lack of healthy non-ischemic ATTM- or ES-Cu-treated controls should be considered when interpreting the pharmacological validation results. Moreover, whole-kidney qPCR cannot fully resolve cell-type-specific expression changes. Finally, the single-cell functional state and CellChat analyses were computationally inferred, and the present experimental validation mainly assessed expression-level associations. Therefore, further cell-type-specific and mechanistic studies, including CRDEG perturbation and rescue experiments, are required to clarify whether these genes directly regulate cuproptosis or cuproptosis-related signaling processes in KIRI.

## 5 Conclusion

We combined bioinformatics analysis, machine-learning approaches, single-cell analysis, and preliminary experimental validation to elucidate cuproptosis-related molecular features in KIRI. We identified four CRDEGs associated with the immune response, oxidative stress, apoptosis-related processes, and renal injury. The four-gene diagnostic model showed good discriminative performance, but requires further validation in larger independent and prospective cohorts. Single-cell and animal analyses further supported the potential involvement of cuproptosis-related stress responses in KIRI, and predicted miRNA/TF regulatory networks and drug‒gene interactions provided exploratory candidates for future studies. Overall, these findings provide a basis for further investigation of CRDEGs as potential biomarkers and therapeutic targets in KIRI.

## Data Availability

Publicly available datasets were analyzed in this study. This data can be found at: https://www.ncbi.nlm.nih.gov/geo/query/acc.cgi?acc=GSE43974, https://www.ncbi.nlm.nih.gov/geo/query/acc.cgi?acc=GSE126805, https://www.ncbi.nlm.nih.gov/geo/query/acc.cgi?acc=GSE161201. All data supporting the findings is available from the corresponding author upon reasonable request.
