# Supplementary figures and images for: Integrative analysis of cuproptosis in kidney ischemia-reperfusion injury: biomarker discovery and diagnostic model construction
Source: Front Mol Biosci. 2026 Jun 26;13:1740289. doi: 10.3389/fmolb.2026.1740289 (PMC13349827; doi:10.3389/fmolb.2026.1740289)

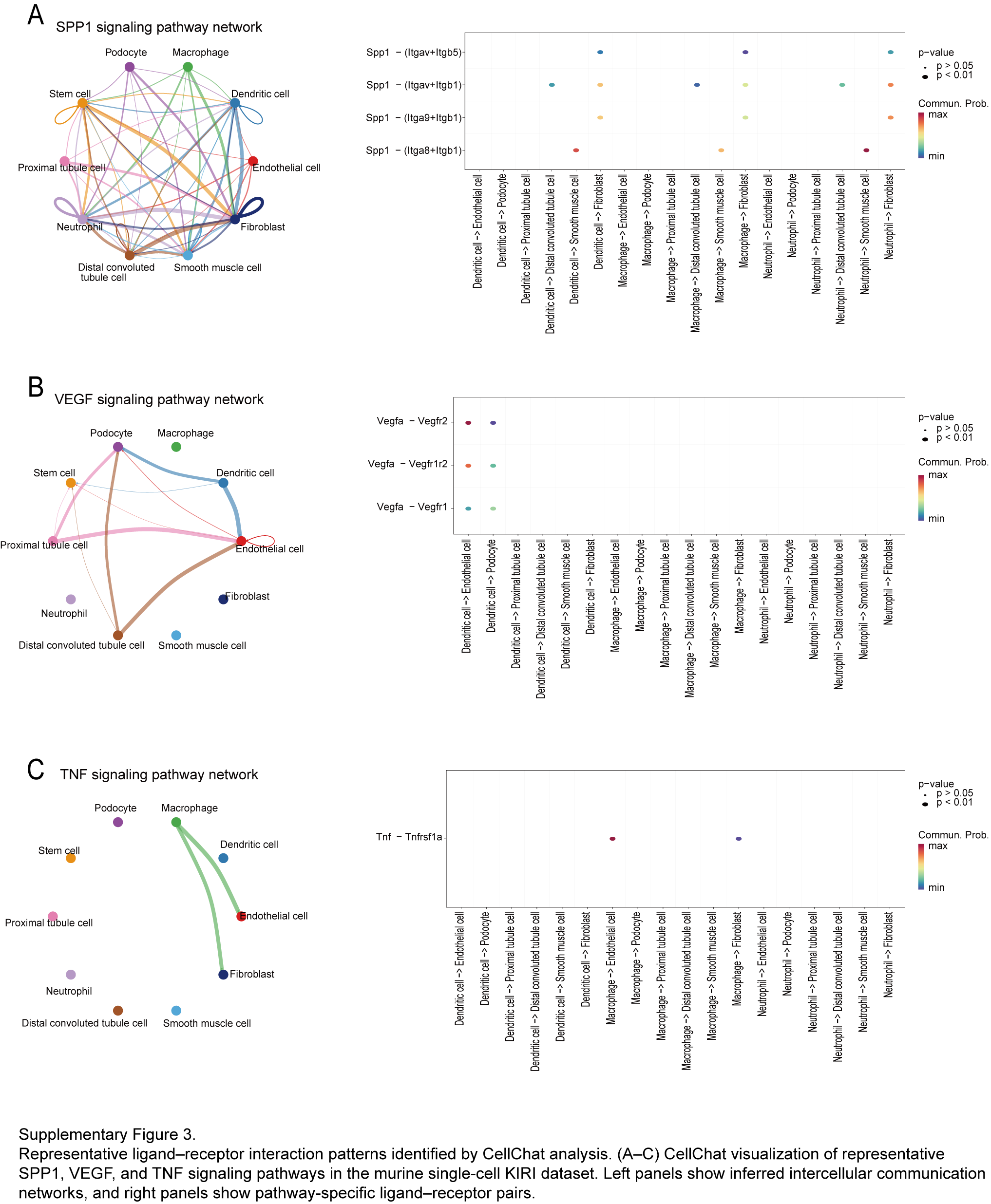

Supplement: Supplementary file 1 [file Image3.tif]

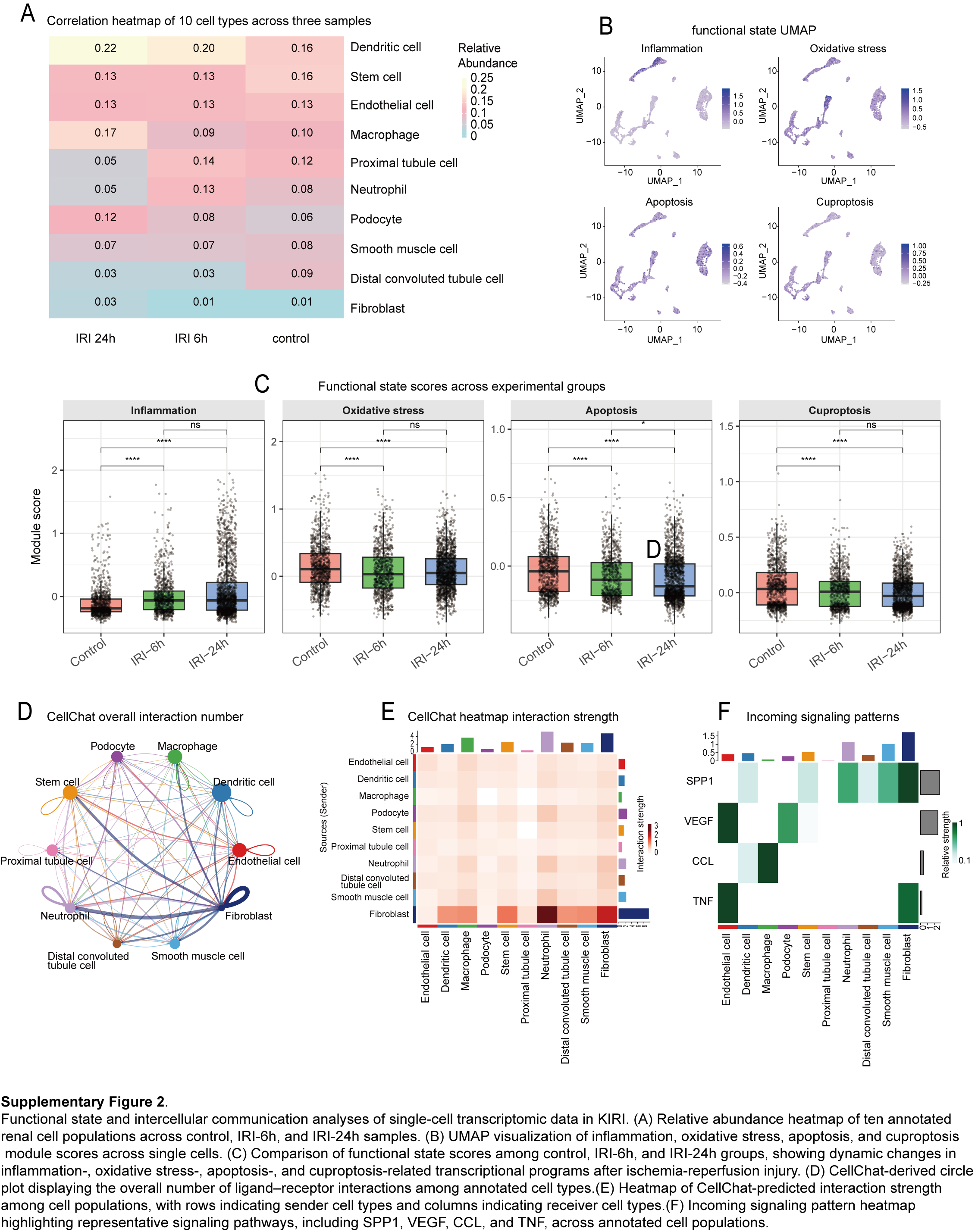

Supplement: Supplementary file 2 [file Image2.tif]

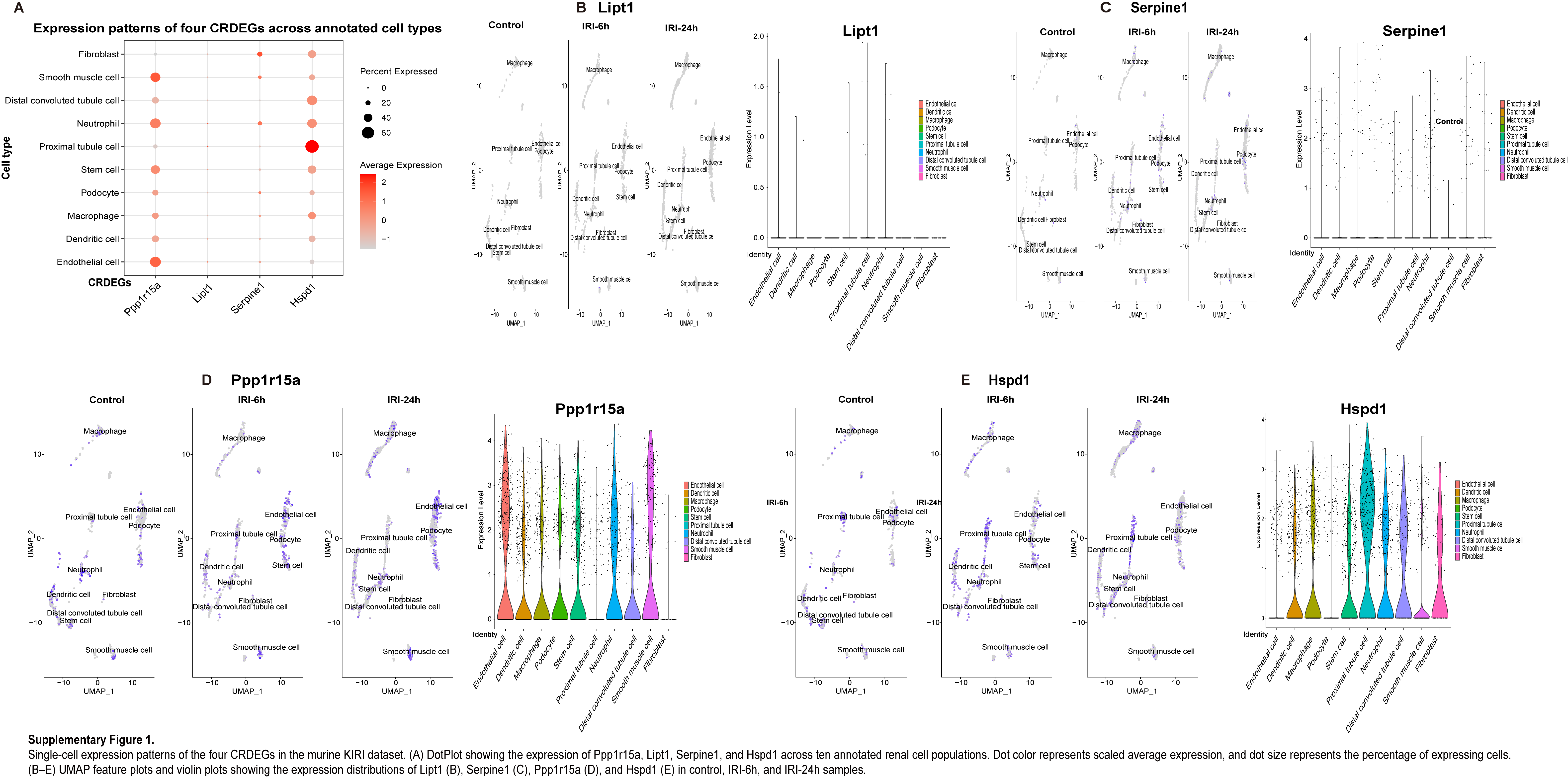

Supplement: Supplementary file 3 [file Image1.tif]
